# Pattern-of-failure and salvage treatment analysis after chemoradiotherapy for inoperable stage III non-small cell lung cancer

**DOI:** 10.1186/s13014-020-01590-8

**Published:** 2020-06-09

**Authors:** Julian Taugner, Chukwuka Eze, Lukas Käsmann, Olarn Roengvoraphoj, Kathrin Gennen, Monika Karin, Oleg Petrukhnov, Amanda Tufman, Claus Belka, Farkhad Manapov

**Affiliations:** 1 Department of Radiation Oncology, University Hospital, LMU Munich, Munich, Germany; 2 Comprehensive Pneumology Center Munich (CPC-M), German Center for Lung Research (DZL), Munich, Germany; 3 German Cancer Consortium (DKTK), Munich, Germany; 4 Division of Respiratory Medicine and Thoracic Oncology, Department of Internal Medicine V, Thoracic Oncology Centre Munich, Ludwig-Maximilians Universität München, Munich, Germany

## Abstract

**Background:**

Loco-regional and distant failure are common in inoperable stage III non small-cell lung cancer (NSCLC) after chemoradiotherapy (CRT). However, there is limited real-world data on failure pattern, patient prognosis and salvage options.

**Methods:**

We analysed 99 consecutive patients with inoperable stage III NSCLC treated with CRT between 2011 and 2016. Follow up CT scans from date of the first-site failure were matched with the delivered radiation treatment plans. Intra-thoracic loco-regional relapse was defined as in-field (IFR) vs. out-of-field recurrence (OFR) [in- vs. outside 50Gy isodose line in the involved lung], respectively. Extracranial distant (DMs) and brain metastases (BMs) as first site of recurrence were also evaluated. Using the Kaplan-Meier method, impact of salvage surgery (sS), radiotherapy (sRT), chemotherapy (sCT) and immunotherapy (sIO) on patient survival was assessed.

**Results:**

Median follow-up was 60.0 months. Median PFS from the end of CRT for the entire cohort was 7.5 (95% CI: 6.0–9.0 months) months. Twenty-six (26%) and 25 (25%) patients developed IFR and OFR. Median time to diagnosis of IFR and OFR was 7.2 and 6.2 months. In the entire cohort, onset of IFR and OFR did not influence patient outcome. However, in 73 (74%) patients who survived longer than 12 months after initial diagnosis, IFR was a significant negative prognostic factor with a median survival of 19.3 vs 40.0 months (*p* < 0.001). No patients with IFR underwent sS and/or sRT. 18 (70%) and 5 (19%) patients with IFR underwent sCT and sIO. Three (12%) patients with OFR underwent sS and are still alive with 3-year survival rate of 100%. 5 (20%) patients with OFR underwent sRT with a median survival of 71.2 vs 19.1 months (*p* = 0.014). Four (16%) patients with OFR received sIO with a numerical survival benefit (64.6 vs. 26.4 months, *p* = 0.222).

DMs and BMs were detected in 27 (27%) and 16 (16%) patients after median time of 5.8 and 5.13 months. Both had no impact on patient outcome in the entire cohort. However, patients with more than three BMs showed significantly poor OS (9.3 vs 26.0 months; *p* = 0.012).

**Conclusions:**

After completion of CRT, IFR was a negative prognostic factor in those patients, who survived longer than 12 months after initial diagnosis. Patients with OFR benefit significantly from salvage local treatment. Patients with more than three BMs as first site of failure had a significantly inferior outcome.

## Introduction

In inoperable stage III non–small-cell lung cancer (NSCLC) the majority of patients will face loco-regional and/or distant recurrences in the first 2 years after the end of primary treatment [1–7]. Intensified follow-up, including Computed tomography (CT) and ^18^F-fluorodeoxyglucose-Positron-emission tomography (FDG-PET) -CT imaging, may lead to a significantly faster detection of asymptomatic disease progression after completed CRT and potentially improve post-recurrence survival [8–11]. Progression after primary treatment correlates strongly with a significant decrease in patients quality of life and survival [12–16]. As shown previously, time to loco-regional recurrence and DMs and their location will also significantly affect patient prognosis [17].

Nowadays, multiple treatment modalities can be offered as salvage therapy, depending on the timing and site of recurrence including local options such as surgery and radiotherapy and systemic therapies i.e. chemotherapy, immune check-point inhibition and tyrosine kinase inhibition [18–26].

To analyse first-site failure pattern and salvage treatment in inoperable stage III NSCLC after CRT, we retrospectively reviewed the medical charts of consecutive patients treated with definitive CRT from 2011 to 2016 at our department.

## Patients and methods

We collected and retrospectively analysed data of 99 consecutive patients, with UICC 7th edition stage IIIA/B NSCLC, treated with curative-intent, multimodal therapy including radiotherapy, at a single tertiary cancer center. All patients were treated between 2011 and 2016, prior to approval of consolidation durvalumab after platinum-based CRT based on the results of the PACIFIC trial. All patients gave written informed consent for treatment and the use of the acquired data for research purposes. This analysis was granted approval by the institutional review board.

Pre-treatment evaluation included radiographic imaging with computed tomography (CT) for all patients, positron emission tomography (PET)-CT in 94% of patients. Cranial contrast-enhanced magnetic resonance imaging (MRI) was performed in 28 patients before the start of multimodal treatment, all other patients received contrast-enhanced head CT.

Tumor histology was obtained in all patients by endo- or transbronchial biopsy (80 patients), CT-guided-biopsy (9 patients) or mediastinoscopy (10 patients). Each individual case was discussed at the multidisciplinary tumor board prior to treatment initiation.

All patients had ECOG 0 or 1, other patients were excluded. Lung function was assessed in all patients before and at the first follow up after treatment completion.

Patients with recurrent disease or with another neoplasia at initial diagnosis, as well as patients who underwent surgery before irradiation, were excluded from this analysis. Cut-off date for the current analysis was July 2019.

Radiation treatment planning and delivery were performed at a single institution, based on PET-CT in treatment position and conventional planning-CT-scans. Radiotherapy was delivered to the primary tumor and involved lymph nodes to a median total dose of 66 Gy (range 50-70Gy). Elective nodal irradiation (ENI) included directly adjacent nodal stations with a total dose of 45–54 Gy in 85% of patients. Radiotherapy was delivered on a linear accelerator (LINAC) with megavoltage capability (6–15 MV) using 3D-CRT in 60% of patients and Intensity-modulated radiotherapy (IMRT) in 40% of patients. Image-guidance was performed with cone-beam CT twice per week.

For the first 2 years after therapy, all patients underwent CT or PET-CT scans, routine blood work, lung function testing and clinical examination every 3 months, every 6 months in the two following years and yearly from the fifth year onwards. Contrast-enhanced MRI of the brain and bone-scintigraphy were performed if clinically indicated.

Intrathoracic recurrences and new distant metastases (DM) were documented with CT, PET-CT and MRI scans. Histological or cytological verification of progressive disease was not obligatory. Scans from date of first-site failure were fused with the delivered treatment plans. Intrathoracic recurrences in the same lung were classified as in-field recurrence (IFR) if within the 50Gy isodose line and out-of-field recurrence (OFR) if outside the 50 Gy isodose line. We evaluated brain metastasis (BM) and extracranial distant metastases (DM) separately. Overall survival was calculated from initial diagnosis. Progression-free survival (PFS) was calculated from the last day of CRT.

Treatments of recurrent and/or progressive disease were tracked in the database.

To minimize the impact of treatment toxicity and comorbidity on survival results, we calculated OS for all patients and for those who survived at least 12 months after initial diagnosis.

Using the Kaplan-Meier method with log-rank test for univariate analysis, the effect of salvage surgery (sS), radiotherapy (sRT), chemotherapy (sCT) and immunotherapy (sIO) consisting of checkpoint inhibitor therapy or tyrosine kinase inhibitor (TKI) on overall survival (OS) was evaluated. We also evaluated post progression survival.

Statistical analysis was performed with IBM SPSS version 25 (Armonk, New York, United States Of America).

## Results

### Patients and multimodal treatment

The majority (63%) of treated patients were males; 56% patients had stage IIIB (UICC 7th edition) disease; the majority had T-stage 3 (30%) or 4 (41%) and N-stage 2 (36%) or 3 (45%). Adenocarcinoma was diagnosed in 50%, squamous cell carcinoma in 42% and NOS in 8% of patients. Analysis of oncogenic driver mutations was performed in 35 patients (70% of patients with adenocarcinoma), 3 (9%) had a mutation in the EGFR gene. ECOG-PS before treatment was 0 in 48 patients and 1 in 51 patients. For the entire cohort median FEV1 was 2.2 l/81% of predicted (range 1.05–3.79 l); median predicted DLCO was 57% (range: 22–93). Median weight loss during treatment was 2 kg. The majority of patients were treated with concurrent CRT to a total dose ≥60Gy (78%). Fifty-two (53%) patients received platinum-based induction chemotherapy. Most Patients (89%) were treated with concurrent or sequential chemoradiotherapy and 11% of patients were treated with radiotherapy alone. Eighty percent of patients receiving concurrent chemotherapy were administered two cycles of Cisplatin 20 mg/m^2^ d1–4 and Vinorelbine 50 mg/m^2^ d1,8,15.. CRT was completed as planned in 95% of patients.

The median follow-up for the entire cohort was 60.0 months (range: 3.8–96.0 months), after initial diagnosis. Median OS for the entire cohort was 20.8 months (95% confidence interval (CI): 15.3–26.3) with one- and two-year survival rates of 76 and 45%, respectively. Patient characteristics are displayed in Table 1.

**Table 1:** Patient, tumor an treatment characteristics

	All Patients
**N**	**99 (%)**
**Age, years**	
median	67.4
range	43–88
**Gender**	
male	62 (63%)
female	37 (37%)
**Tobacco consumption**	
median PY	40
range	0–150
**Atelectasis before RT**	10 (10%)
**Tumor Histology**	
Adenocarcinoma	49 (50%)
SCC	42 (42%)
NOS	8 (8%)
**ECOG performance status**	
0	48 (48%)
1	51 (52%)
**Lung function testing**	
Fev1 median (range)	2.2 l/81% (1.05–3.79 l)
DLCO	57% (range: 22–93)
**UICC 7th edition Stage**	
IIIA	44 (44%)
IIIB	55 (56%)
**T-Stage (UICC 7th edition)**	
unknown	2 (2%)
1	10 (10%)
2	17 (17%)
3	30 (30%)
4	40 (41%)
**N-Stage (UICC 7th edition)**	
0	10 (10%)
1	9 (9%)
2	36 (36%)
3	44 (45%)
**Tumor localisation**	
central	40 (40%)
pancoast	7 (7%)
lobular	52 (53%)
**PET-CT**	
before CRT	93 (94%)
after CRT	35 (35%)
**Gross tumor volume (ccm)**	
mean	109.9
median	85.3
range	3–434
**Chemotherapy**	
Concurrent or sequential CRT	88 (89%)
Induction chemotherapy	52 (53%)
Concurrent chemotherapy	77 (78%)
**Radiation Technique**	
3D-conformal	59 (60%)
IMRT	40 (40%)
**Total Dose**	
Mean	62.4Gy
Median	66Gy
< 54	13 (13%)
54.01–60	19 (19%)
60.01–66	58 (59%)
> 66.01	9 (9%)
**CRT completed as planned**	94 (95%)

### First-site failure pattern

For all patients median PFS was 7.5 months (CI: 6.0–9.0 months) from end of CRT**.** Six, 12-, 18- and 24-month PFS rates were 60, 30, 21 and 15%, respectively. Fifty-one (52%) patients developed intra-thoracic loco-regional recurrence; of which 26 cases (26%) were defined as IFR and 25 (25%) as OFR, respectively**.** DMs and BMs as first site of failure were detected in 27 (27%) and 16 (16%) patients, respectively**.** A chronological distribution of the first-site failure after end of CRT is documented in Table 2.

**Table 2:** timing of IFR, OFR, DM and BM

	median time in months	range in months	6 months rate	12 months rate	24 months rate
IFR	7.2	1.5–46.6	7%	21%	25%
OFR	6.2	0.6–57.9	11%	17%	22%
BM	5.1	1.1–28.3	8%	13%	15%
DM	5.8	0.4–42.7	14%	22%	24%

No survival difference in patients receiving 3D-CRT (23.1 months) vs. IMRT (18.3 months; *p* = 0.375), nor difference in PFS in patients receiving 3D-CRT (7.7 months) vs. IMRT (6.5 months; *p* = 0.292) was observed.

### Intra-thoracic in-field recurrence (IFR)

**Fig. 1:**
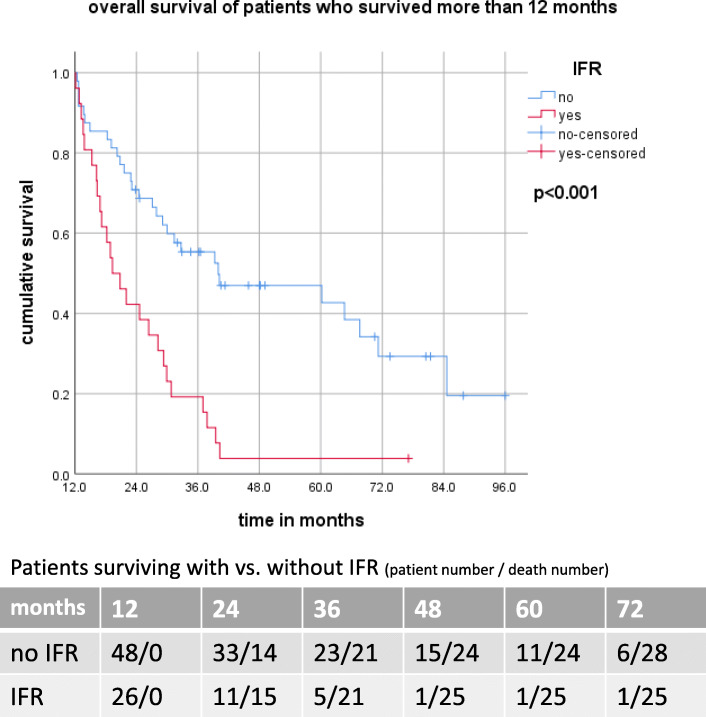
OS with vs without IFR for patients who survived longer than 12 months

Twenty-six patients (26%) had IFR, of which 7 (27%) occurred within the first 6 months, 21 (81%) within 12 months and 25 (96%) within 24 months after the end of CRT. Median time to IFR was 7.2 (range: 1.5–46.6) months. Sixteen (27%) patients treated with 3D-CRT presented with in-field recurrences after a median interval of 8 months, whereas 10 (25%) patients treated with IMRT developed in-field-recurrences after a median of 6.4 months. IFR had no significant impact on overall survival (19.3 vs. 22.9 months; *p* = 0.151) in the entire cohort. However, IFR was a significant negative prognostic factor in patients who survived longer than 12 months after initial diagnosis (19.3 vs 40.0 months; *p* < 0.001). (Fig. 1).

No patients with IFR received sS or sRT. Eighteen (70%) patients underwent sCT with no OS difference (*p* = 0.742). Five (19%) patients received sIO with mOS of 33.6 vs. 31.4 months (*p* = 0.921), respectively.

### Intra-thoracic out-of-field recurrence (OFR)

Twenty-five patients (25%) developed OFR with no significant impact on OS (27.1 vs 20.8 months; *p* = 0.313) in the entire cohort and in patients who survived longer than 12 months from initial diagnosis (64.6 vs 28.2 months; *p* = 0.214). Median time to OFR was 6.2 (range: 0.6–57.9) months. OFR occurred in 11 (44%), 17 (68%) and 22 (88%) patients 6, 12 and 24 months after the end of CRT.

Three patients (15%) were treated with sS and survived until the time of cut-off (36 months survival rate 100%). sRT was performed in five (20%) patients and associated with a significant survival benefit (72.4 vs 19.1 months, (*p* = 0.014).

Thirteen (52%) patients with OFR underwent sCT (OS: 26.4 vs 32.7 months; *p* = 0.644). Four (16%) patients received sIO with pronounced survival benefit (mOS: 64.6 vs. 26.4 months; *p* = 0.222).

### Brain metastases (BMs)

Sixteen patients (16%) developed BMs as first site of failure after CRT. Median time to diagnosis of BMs was 5.1 (range: 1.1–28.3) months; 8 (50%), 13 (81%) and 15 (94%) patients were diagnosed with intracranial relapse 6, 12 and 24 months after the end of CRT. Twenty-eight (28%) patients had contrast-enhanced MRI of the brain before the start of CRT; four (14%) developed brain relapse thereafter. In patients with contrast-enhanced cranial CT before CRT, 12 patients (17%) developed brain metastases. The time to diagnosis of brain metastases and the OS was not different in MRI vs. CT patients. BMs accounted for 38% of all distant recurrences and had no impact on survival in the entire cohort (19.1 vs 20.8 months; *p* = 0.635) and in patients surviving longer than 12 months after initial diagnosis (31.4 vs 28.2 months *p* = 0.611).

**Fig. 2:**
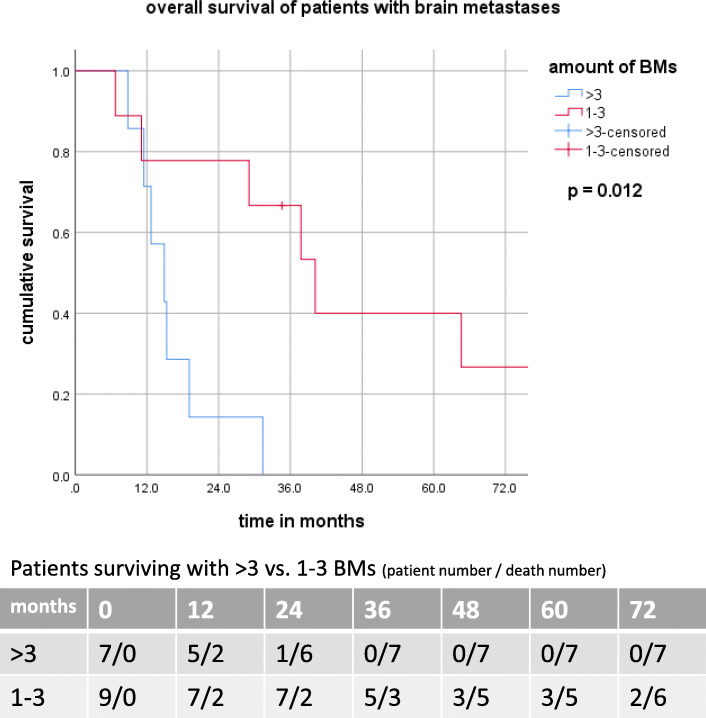
OS with 1-3 vs with > 3 BM

Nine (56%) patients had three or less BMs with mOS of 26.0 vs 9.3 months (*p* = 0.012) compared to those with multiple intracranial lesions (Fig. 2).

Whole brain irradiation was delivered in 7 (47%) and stereotactic radiosurgery (SRS) in 8 (53%) patients with a trend towards improved survival after SRS (mOS 15.3 vs 37.8 months; *p* = 0.064). One patient was not treated and received best supportive care. Additional sCT and sIO had no significant impact on prognosis.

### Extracranial distant metastases (DMs)

Twenty-seven patients (27%) developed extracranial DMs as first site of failure, after a median time of 5.8 (range: 0.4–42.7) months. In the first 6, 12 and 24 months after CRT, DMs were detected in 14 (14%), 22 (21%) and 24 (24%) patients, respectively. Median OS of patients with DMs was 18.2 compared to 22.0 months for the rest of the cohort (*p* = 0.536). DM also had no impact on OS in patients who survived longer than 12 months after initial diagnosis (mOS: 29.9 vs 26.4 months; *p* = 0.939).

Nineteen (19%) patients had in total less than three lesions at time of the first failure. Median overall survival in this subgroup was 21.6 vs 11.0 months (*p* = 0.177) compared with patients with ≥3 metastases. Of these 19 patients, 8 (42%) had bone, 5 (26%) lung, 3 (16%) adrenal gland, 2 (11%) extra-thoracic lymph node and 1 (5%) liver metastasis.

### Post recurrence survival and subsequent failures

Median survival of patients after diagnosis for IFR, OFR, BMs and DMs was 6.9 (95%CI: 3.8–9.9), 7.7 (95%CI: 5.3–10.1), 9.6(95%CI: 2.5–16.8) and 6.4 months (95%CI: 3.5–9.4 months), respectively.

**Fig. 3:**
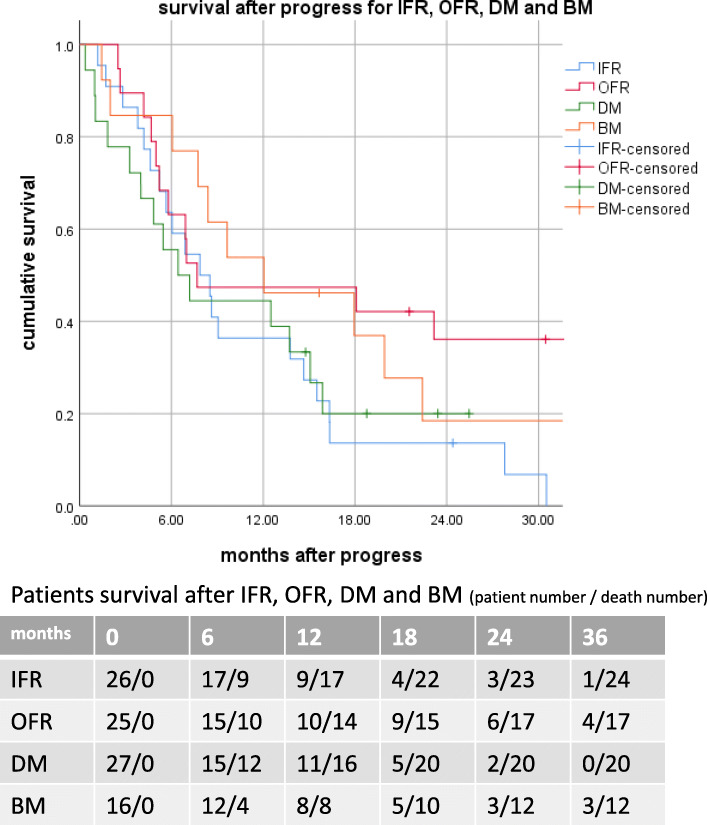
Survival after progression

6-, 12-, 18- and 24 - month survival rates were 65, 35, 15 and 12% for IFR; 60, 42, 38 and 26% for OFR; 75, 50, 33 and 20% for BMs; 55, 40, 20 and 9% for extracranial DMs. Importantly, we found a difference in post-recurrence long-term survival regarding the site of failure, with the most favourable outcome for OFR (Fig. 3).

Out of 26 patients with IFR as first manifestation, we observed subsequent DMs in 6 (23%) and BMs in 2 (8%) patients. Out of 25 patients with OFR first, we observed subsequent DMs in 9 (36%) and BMs in 4 (16%) patients, respectively. In patients with DM first (27), we observed subsequent BMs in 14 patients (52%), whereas loco-regional relapse occurred in 10 patients (37%), respectively. In patients with BM first (16), we observed subsequent DMs in 3 (18%) and loco-regional relapse in 4 patients (25%), respectively.

## Discussion

The aim of the present study was to analyse the pattern of failure as well as salvage treatment of first site failure and its prognostic relevance in patients with inoperable stage III NSCLC after primary CRT from 2011 to 2016. All patients had been treated prior to the approval of consolidation durvalumab after platinum-based CRT based on the results of the PACIFIC trial [6, 7]. The study revealed, that in a majority (70%) of patients, disease progression occurred in the first 12 months after completion of definitive treatment, while median time to intracranial relapse was the shortest. Altogether 94% of patients had local and/or distant tumor progression after completion of primary treatment. An explanation of this phenomenon could be a very long follow up time in the analysed cohort. Additionally, because histological confirmation of intrathoracic recurrence was not required, patients with secondary lung malignancies may have been included. Most patients (51%) experienced intra-thoracic loco-regional recurrence, with two-year IFR and OFR rates of 25 and 22%, respectively. Over time, the numbers of cases with intra-thoracic recurrence continuously increased, with IFR rate rising significantly from 7% at 6 to 25% at 24 months after the end of CRT. In contrast, BMs as first site of failure were detected in 8% of patients at 6 and 15% at 24 months after CRT**.**

Our results regarding rates and timing of first-site recurrence are in accordance with a study reported by Grass et al. with a one-year disease progression rate of 74% compared to 70% in our cohort. Also, the relapse pattern was comparable in both studies: 27, 29 and 55% in the study by Grass et al. vs. 26, 25 and 43% for local, regional and distant recurrences in the present analysis, respectively. Grass et al. also demonstrated that symptomatic relapse leads to significantly inferior patient outcome, compared to relapse detected with routine aftercare imaging [17]. A recent report from Bodor et al. did not detect this difference, but revealed that occurrence of one to three BMs as first-site of failure was associated with a significantly longer survival compared to the presence of multiple BMs [27]**.** This is also in close agreement with our findings, with a median survival of 26.0 vs 9.3 months after development of three or less vs. multiple BMs, respectively. It is important to note that the low rate of cranial MRIs performed before treatment in this cohort might have resulted in the potential inclusion of patients with undetected BMs.

A relevant finding of present analysis is a strong negative impact of IFR as first site of failure in patients who survived longer than 12 months after initial diagnosis, with a median survival of 19.3 vs. 40.0 months for patients with and without IFR, respectively. Furthermore, this impact of IFR was not reproducible in the entire cohort. This finding stressed a prognostic importance of timing of disease progression after CRT. While not detecting significant OS differences between patients with OFR, BMs and DMs compared to the entire cohort, the present study reveals an important difference concerning post-recurrence long-term survival. Patients with OFR and BMs as first site of failure achieved significantly higher 18- and 24-month survival rates compared to patients with IFR and DMs. This phenomenon may be explained by more effective salvage treatment. In our study patients with OFR as well as patients with three or less BMs benefited mostly from ablative therapy, i.e. sRT or sS. Also, sIO was associated with improved survival in patients with OFR.

Salvage treatment for stage III NSCLC treated with definitive CRT has dramatically changed in the last decade. Development of image-guided, high precision re-irradiation protocols, especially particle re-irradiation as well as introduction of immune check-point inhibition and chemoimmunotherapy have led to continuous improvement of post-recurrence survival [28–32]. Intensive aftercare imaging programs have also significantly influenced post-progression patient outcome [33].

Nevertheless, loco-regional recurrence is still difficult to manage, especially if relapse occurs within the intensively pre-treated area (IFR) [34]. It is important to note that no patient with IFR was re-irradiated or had salvage surgery in the present study**.** We also detected no survival benefit for IFR patients receiving sCT or sIO. Schlampp et al. recently reported a lung cancer patient median survival, following re-irradiation, of 9.3 months and a local progression-free survival of 6.5 months, a considerable improvement to 6.9 months post-recurrence survival in our cohort [35].

In contrast, for patients with OFR, local ablative treatment is more reasonable and should be considered. Our study confirmed the previously described role of ablative salvage treatment in patients with OFR [29, 36–44].

Concerning DMs, oligoprogression, defined as progression with less than three lesions, was the dominant recurrence pattern in the present study. Patients with oligoprogression had a significantly longer survival compared to patients with multiple metastases. This is also in accordance with previous data [45–47] .

Furthermore, pattern of failure analysis after CRT is important in terms of the reported excellent disease control rate and long-term outcome in the first randomized phase III chemoradioimmunotherapy trial for locally-advanced NSCLC (PACIFIC). It remains however to be seen if failure patterns will significantly change with the recent implementation of the consolidation therapy with durvalumab after completion of CRT, especially concerning BMs, which appear to be less frequent [6, 7].

Acknowledging the limitations of the present study, it is important to note, that this is a retrospective analysis based on data from a single tertiary cancer center. Nevertheless, the present results are in close accordance with previous reports and comprehensively characterize real-life disease progression patterns as well as delivered salvage treatment and corresponding patient outcome.

## Conclusion

The present study provides an overview of the real-life failure pattern and delivered salvage treatment in inoperable stage III NSCLC after completion of CRT prior to PACIFIC. The study suggests that it is important to differentiate between IFR and OFR. Treatment and survival of patients with IFR remain limited and challenging and should be further intensively investigated in prospective studies.

In contrast, patients with OFR benefit significantly from ablative salvage treatment options, which could be further reinforced with sIO.

## Data Availability

The datasets used and analysed during the current study are available from the corresponding author on reasonable request.
